# Practical Observations on Aural Surgery and the Nature and Treatment of Diseases of the Ear

**Published:** 1855-01

**Authors:** 


ART. III.—*Practical Observations on Aural Surgery and the Nature and Treatment of Diseases of the Ear.* With Illustrations. By WILLIAM R. WILDE; Fellow of the Royal College of Surgeons in Ireland; Surgeon to St. Mark's Ophthalmic Hospital. Philadelphia: Blanchard & Lea. 1853. pp. 475, 8vo.

Although some months have elapsed since the first appearance on this side of the Atlantic, of the work which forms the caption of this article, we have never found time to notice it in a manner befitting the importance of its contents or the reputation of its author.

If any one will take the pains to look over the periodic and "manual" literature of the day he cannot fail to be struck with the singular and deplorable absence of everything relating to the history, symptoms, causes, mode of treatment, and results of the most frequent and remarkable diseases of the Ear.

With here and there an exception the modern systems of surgery contain but scanty information on the subject, and students, with perhaps still rarer exceptions, continue to be taught theoretically to believe, and practically to observe, that we "know nothing" about the diseases of the organs of hearing.

We are acquainted—there is not one of our readers who is not—with medical men, most astute and practical physicians and surgeons in all other respects, who treat diseases of the ear by prescribing nostrums, of both a local and general character, which they would never think of using in similar forms of disease in any of the other organs of the body.

We will not stop here to enquire into the causes which produce and continue this species of empiricism further than to ask, if it does not mainly arise from the want of proper attention to the subject in our schools? How many of the surgical instructors, the "heads of the profession," in this country, devote more than two hours annually to the consideration of aural affcetions? In how many of our medical colleges are diseases of the ear never even hinted at? They are *taught* in none. They are blinked at and given the go-by in all, while diseases of the nails, inflammation of the urethra, harmless little tumors and other like affections are dwelt upon eloquently and at great length.

We confess, in the outset, that the study of the anatomy of the ear is difficult. The organ itself is small. Its depth, the complexity of its structure and the small hard bone in which it is situated render its dissection laborious, difficult, and very tedious. The number of crabbed names attached to its different parts by no means adds to its attractiveness, and the way in which anatomical demonstrations are usually conducted contributes but little to its interest.

Yet the anatomy of the ear must be understood before its diseases can be properly studied. Its physiological appearances must be seen and learned before its pathological changes can be appreciated or remedied.

There are diseases of the outer ear—of the middle ear—of the inner ear, and before these can be recognized or treated with any rational hope of success, it is indispensable that the physician should know the anatomical boundaries of these several chambers and the appearances that they present in health.

In this study, as in many others, the practitioner must be his own master. He must read and dissect for himself. He must explore the ears of living men for himself. This he should do with a two fold object, first, to familiarize himself with the natural, healthy aspect of the parts, and secondly, in order to acquire that dexterity of manipulation which comes almost alone from practice, and which is so indispensable to either a satisfactory examination of the organ or the performance of any operative procedures. He should add an ear speculum to his pocket case. It is by the aid of the polished surfaces of this little funnel-shaped instrument that those portions of the ear most subject to disease are brought within his view. The ear speculum has done for the organs of hearing what the uterine speculum has done for the uterus. Without it, we would still be working in the dark. With it, the progress which this branch of medical science is making is in all probability as rapid as that in any other department of the healing art. With it, literally, a flood of light has been thrown upon aural diseases.

But to pass on. The first chapter in Mr. WILDE's work is devoted to introductory remarks and the bibliography of aural surgery. He traces the latter subject from HIPPOCRATES down to the famous Dr. TURNBULL, of London. Of all the long list of authors whether ancient or modern, who have written on the ear and its diseases, it is the opinion of Mr. WILDE that the labors and investigations of Mr. TOYNBEE are of more practical value than those of all his predecessors either in England or the Continent.

He says, "Mr. TOYNBEE's researches prove the position which I long ago advanced, and which, from year to year, I have been in the habit, not only of teaching theoretically, but practically demonstrating in my clinical lectures, that the great majority of diseases of the ear producing deafness have their origin in inflammation of one kind or another."

It is to be regretted that Mr. TOYNBEE has not yet published any separate work on the ear, but has confined himself to contributions to the periodicals of Great Britain.

In order that our readers may see what has been done in this country in the way of contributions to aural pathology, we will quote Mr. WILDE on that subject: "I am not acquainted" he writes, "with any native American work on aural surgery; and the medical periodicals of that country have recorded but few cases of interest in connection therewith. The only book which I know is that of SAISSY, 'An Essay on the Diseases of the Internal Ear, translated from the French by NATHAN R. SMITH;' Baltimore, 1829. The first volume of Baron LARREY's '*Clinique Chirurgicale*, containing a chapter on Lesions of the Ear, was translated by Dr. RIVINUS, of Philadelphia, in 1832.' The American editor, Dr. HEWSON, of Philadelphia, who was a pupil of Mr. WILDE, has added the following note on this subject. 'Dr. SMITH's translation contains a supplement of seventeen pages octavo, by him, on *Diseases of the Ear*, and *a description of* his *instrument for perforating the membrana tympani.*' Besides the above, there is an American edition of PILCHER, with notes (chiefly confined to the pathology of hearing), published by Barrington & Haswell, Philadelphia, 1843.

"There was a reprint, in 1838, of Dr. BENNETT's translation of KRAMER, by Thomas, Cowperthwait & Co., of Philadelphia, and one of DUFTON's book by Lea & Blanchard, in 1848. There was a small duodecimo of twenty-four pages, entitled 'A Treatise on the Anatomy, Physiology and Diseases of the Ear,' by JAMES BRYAN, M. D., of Philadelphia, published by the author in 1851."

These constitute the sum total of the labors, in this direction, of American authors, and the works to which American students have had access.

In chapter 2nd. *The means of diagnosis, and application of remedies* are dwelt upon at a most satisfactory length and with far more minuteness than is usual in works of this description.

In order to arrive at accuracy of diagnosis—the first grand point in all diseases, but especially in aural affections, and without which all treatment must be empirical,—it is indispensably necessary that we should be thoroughly acquainted with the best mode of conducting an examination. How often has the practitioner, when consulted by an individual suffering from ear disease, anxiously enquired what that "best mode" is. Here we have it in Mr. WILDE's own words:

"The patient being placed opposite strong, direct sunlight, with the head inclined at such an angle that the sun's rays may fall directly through a tubular speculum upon the membrana tympani, we first carefully observe the condition of the concha, external meatus, mastoid process, infrazygomatic region, and the space immediately below the lobe of the ear. The *auricle*, in its various folds, its color, its temperature in particular, its thickness as learned by feeling its hem or helix between the fingers, and the angle which it forms posteriorly with the cranium—together with the position, size, shape, and color of the *external meatus*, as seen without altering the relation of the parts—should be specially noticed. The upper rim of the helix should then be grasped between the finger and thumb of one hand, and drawn upwards, backwards, and outwards, while the thumb of the other hand, placed in front of the tragus, by drawing it and the integuments forward upon the zygoma, exposes the outer third or more of the auditory canal to view. The finger should then be pressed deeply and firmly upon the moveable root of the *tragus*, and backwards into the depression between it and the articulation of the jaw. While the finger is retained in this position the patient should be desired to open and shut the mouth, and the amount of pain or inconvenience experienced by pressure in those two different positions of the jaw accurately noted. The middle and fore-fingers should likewise be inserted deeply behind the ramus of the jaw towards the styloid process, and notice taken of the sensations there experienced.

"Where we have reason to believe inflammatory action exists, the *mastoid process* in an especial manner claims our attention. Its color, size, shape, and temperature, may be learned by even a cursory examination; but, besides this, it should be most carefully pressed upon with a couple of fingers, with a much greater degree of force and firmness than is usual in making examinations of the the like nature elsewhere; and this examination should not only be applied to the mastoid region, but to the whole posterior and lateral portion of the head, if we have reason to suspect any inflammation, or its effects. The insertion of the sterno-mastoid, as well as the upper third of that muscle, should also be carefully examined in the same way, as there is a small gland, in shape and size like a horse-bean, situated immediately behind the auricle, over the middle of the mastoid process, which frequently becomes enlarged during the progress of aural inflammations, and is also the seat of violent neuralgic pain in some instances. If the integuments and soft parts are swollen or œdematous, as is frequently the case in certain inflammatory affections of the ear, as also where they have become thickened from long continued disease, it will require a considerable degree of force to make a perfectly satisfactory examination. The amount of pitting made by the finger during this examination and its degree of permanency, are also circumstances of value in the formation of a diagnosis. Percussion of the mastoid process, immediately behind the attachment of the auricle, occasionally affords some information, as will be shown in some of the cases hereafter detailed.

"We next proceed to inquire into the condition of the *auditory canal*, and external surface of the membrana tympani. To effect this, and to explore every portion of the surface of these parts, it is necessary to resort to the mechanical assistance of the speculum; first taking care to remove any impaction of wax, accumulated discharge, or other mechanical impediment which may exist and obstruct our vision. If this obstruction is complete, and we have reason to suppose that it is the chief cause of deafness, the employment of a syringe and some plain warm water is the best mode of removing it; but if the obstruction merely co-exists with other, and particularly with some of the inflammatory affections of the meatus or tympanal membrane, or if it be only partial, and consists of portions of detached cuticle, hairs, or scales of hardened, inspissated cerumen, it is better to remove these gently with a pair of fine forceps, because the very act of syringing, even with warm water, causes in a healthy ear an increased vascularity, which will mask the actual amount of disease present. The same observation applies also with respect to slight otorrhœa, but if there be much discharge present, we must have recourse to the syringe."

Mr. Wilde discards all speculi except the tubular. This little instrument is a simple conical silver tube, one inch and a half long, five-eighths of an inch wide at the greater aperture and varying from two to four lines in the clear at the smaller extremity. Whatever instrument is used, "the patient should be seated beneath the examiner, with the head slightly bent, opposite a window *through which the sun is shining at the moment*, and, if possible, between the hours of eleven and three."

Various instruments producing a ticking sound by means of clockwork, have been invented by Schwalz and others, for the purpose of ascertaining the *degree of deafness* and the *hearing distance*, but none, it appears, answer so well as an ordinary watch.

Whatever instrument, however, is used, the fact should not be lost sight of that there is almost as great a difference in the normal hearing as there is in the normal seeing distance, even among persons who have never labored under any ear disease, and who are not at all conscious of any defect of hearing.

It is also important to inquire whether the hearing is better at one hour of the day than another—whether it is augmented or diminished after meals, particularly dinner—whether improved or not when the individual is exposed to loud noises, as when standing in a mill, travelling in a carriage &c. In the next place the *throat*, the back of the pharynx, the uvula, tonsils and arches of the palate should be examined as regards the condition of the mucous membrane—its color, turgescence, degree of relaxation &c. An important diagnostic sign may be sometimes obtained by the amount of pain excited there and in the middle ear, by the point of the fore-finger being pressed firmly upwards and outwards beyond the arch of the palate, opposite the mouth of the eustachian tube. The state of the membrane of the nose should also be observed.

The voice may furnish valuable aid in the examination, as few cases of intense or long continued deafness occur without that function becoming more or less changed. This defect appears to be independent of any alteration in the parts engaged in the mechanism of speech; it is often observed without enlargement of the tonsils, or other abnormal condition of the mouth, throat or larynx.

The routine of examination that we have described has been demonstrated by experience to be the most practically useful. The history, assigned cause, &c., of the disease and the subjective symptoms are elicited in the usual manner in which we proceed to examine any other surgical or medical case. The value of *tinnitus aurium* as a diagnostic, Mr. WILDE asserts has been greatly overrated. It is, notwithstanding, a symptom which should be carefully inquired into, whether permanent or intermitting; under what circumstances increased or diminished, and especially whether referred to the ears or interior of the head; whether one or both ears are equally affected.

"Ringing in the ears" is one of the most distressing, as well as the most constant symptoms attendant upon affections of the organs of hearing. It may be present in several aural diseases, or it may exist as an isolated symptom—it is common to almost all, and peculiar to none, of the diseases of the ear—its cause is very obscure and difficult to comprehend, and its removal still more difficult to accomplish. It is often caused by cerebral disease. Derangements in any of the great systems, or so slight a cause as simple catarrh, congestions in any important organs, or even so insignificant a foreign body as a hair resting on the tympanal membrane, may produce it. The noise will sometimes disappear spontaneously. Again, it will remain after the original disease has been removed, a healthy action imparted to the affected organ and its function restored.

A tolerably accurate diagnosis can be formed in almost every case if attention be paid to the rules laid down by Mr. Wilde. And this being done, we will no longer hear of persons being cupped and leeched, starved and blistered, purged and salivated for tinnitus, when it may depend, as has been just remarked, on a comparatively thin cake of hardened wax, or a single small hair impinging on the drum of the ear.

The ignorance which prevails with regard to the mode of applying even the ordinary remedies employed in aural diseases, is almost as great as obtains of their pathology. Although most of the affections of the organs of hearing are originally of inflammatory origin it is seldom necessary to resort to general bleeding; but local depletion either by means of cups or leeches is imperatively required. None but a very dexterous cupper will be able to apply his glasses sufficiently near the part affected to be of much service; hence, leeches are the most effectual means of abstracting blood and relieving pain; they should not, however, be applied, as is so generally the custom, behind the mastoid process; they should be attached immediately around and within the edge of the external meatus, in the fossa behind the tragus, and, if necessary, in front of that prominence, in the hollow formed by depressing the jaw. "The external meatus should first be filled with a bit of cotton wool, to a level with the external aperture, not so much for the purpose of preventing the leeches going in too far, as to exclude the blood, which is very likely to flow back and accumulate at the bottom of the meatus auditorius externus, coagulating and crusting over the surface of the tympanal membrane, thereby causing much annoyance to the patient, and even an aggravation of his symptoms. The posterior lip of the external aperture affords the largest and most convenient surface for the application of leeches, and in an adult, three may always be attached thereto with facility. The anterior lip, being more concealed and slightly concave, cannot so well be got at, yet two may generally be applied there. The next best part to which to apply them is the depression in front of the tragus, immediately below the inferior root of the zygoma, where in aural inflammations the patient is so frequently susceptible of pain upon the least pressure, and there, six or eight may be applied if necessary." When the mastoid region becomes the seat of inflammatory action, they should also be applied freely all over it. There is no painful affection in which leeches applied in the manner directed by Mr. WILDE produce the same amount of immediate relief, as in diseases of the ear. "They should be had recourse to again and again, even upon the same day, and applied in numbers, to relieve paroxysms of pain, as well as to lessen the degree of redness and vascularity observable in the inflamed parts."

Counter irritation with the common fly-blister, or, as Dr. HEWSON suggests, with the cantharidal collodion, is beneficial in acute cases. Pustulation with emetic tartar is especially indicated in old chronic cases, where there is much thickening of the tympanal membrane. In children and young persons the strong tincture of iodine, containing some hydriodate of potash, is a useful application. To be effective, it must be used daily or every second day and persisted in for a long time. Should there be much neuralgic pain present, extending from the ear over the side and back of the cranium, the compound camphor liniment, with extract of belladonna will be found an exceedindly useful application.

While fomentations and stupes are not as efficacious in inflammations of the ear as in those of the eye, the application of heat and moisture is generally particularly grateful. A warm flaxseed meal poultice, frequently renewed, gives great comfort; and the vapor of very hot water charged with hyoscyamus, belladonna, opium, or the ordinary decoction of marsh mallows, camomile, or poppy heads, has an exceedingly soothing effect when continuously applied to the ear.

*There are no circumstances which justify the pouring of sedative or stimulating liquors into the ear.* "If there is one substance more irritating than another in the Pharmacopœia, it is poured, *secundum artem*, into the ear to relieve pain, or cure deafness, to lessen or increase the secretion of wax! This practice is often the cause of myringitis. Why are not these essential oils, stimulating liniments, this turpentine, creasote, tincture of cantharides, oil of origanum, &c., poured into the eye, or injected into the urethra, in cases of inflammation of these parts? Why do not surgeons prescribe a roasted onion, or a boiled fig, for inflammations of other parts as well as the ear?"

The usual routine of treatment of aural diseases is very much the same in this country as in Great Britain. First, syringing with hot water and either castile or old brown Windsor soap, then setting the digestive organs to rights by purgatives and "a course of bitters," then blistering behind the ears. These, and such like methods failing to relieve, stimulants, often of the most acrid nature, are poured into the external auditory passages. Then come the pungent essential oils, creasote, turpentine and hot tinctures, either or all of which are mercilessly applied to the external surface of the tympanal membrane. We have before us at this moment a recipe, copied from a recent standard work, composed of laudanum, sulphuric ether and spirits of camphor, which is recommended as an application to the ear in pains of that organ.

Where the practitioner is less bold he may resort to more palliative means and content himself with advising a little warm almond oil to be dropped into the ear at bed time, or Cologne water to be rubbed on the cheek of the diseased side, at the same time enjoining a little black wool to be retained in the meatus, for the purpose of protecting the organ against cold. In order to ensure the full amount of good contained in the latter remedy, the wool must have been plucked from the left fore-foot of a six years old black ram! A slice of fat bacon inserted into the meatus every second night is a highly popular remedy, and if old women and some very respectable practitioners are to be credited, its exhibition is often of great service. Galvanism and electricity are now put on trial—these are followed by glycerine, which may be said to be the fashionable remedy of the day, and if this does not succeed change of air and scene, sea bathing, or "a course of waters" at some of the fashionable places of resort for that purpose, is prescribed. Could there be any plan of treatment more diametrically opposed to all the received opinions of inflammations of other parts of the body, than this we have been describing? And yet how many of us have witnessed its adoption!

The great mass of aural diseases being, in the opinion of our author, the production of inflammation in some form or other, it is apparent that such agents as electro-magnetism, galvinism, or electricity, can effect little for their removal. Mr. WILDE declares that he never knew a case which had proved unamenable to other treatment cured by any of these means.

"Mercury is the medicine which of all others acts most beneficially in diseases of the ear, simply on account of its specific efficacy in arresting, or controlling inflammation or removing its products." It is applicable to a variety of aural affections, is administered in a variety of modes and forms, each specially apposite to the particular stage of the disease, the class of symptoms, or the peculiar habits and constitution of the patient. As a general rule, "the modes of exhibiting it in ocular affections will serve as a safe guide for giving it in diseases of the organ of hearing. In the more violent inflammations of the fibrous structure of the membrana tympani, the periosteal lining of the cavity of the tympanum, the Eustachian tube, or the deeper portion of the external meatus, and also the inflammations of the internal ear, when such can be diagnosed, as well as the specific inflammations of a rheumatic or syphilitic character, where actual ptyalism is indicated, we must introduce it quickly, and in such doses as will bring the system under its influence at once, just as we would in inflammations of analogous tissues in the eye, the envelopes of bones, or the membranes investing the joints or any of the great cavities of the body. In such cases small and frequently repeated doses of blue pill and calomel, with opium, will most speedily produce the desired effect, provided the well known rules for the administration of mercury are attended to."

In chronic or subacute forms of aural disease mercury is very precious either as an alterative, or to keep up sustained but gentle action on the mouth, and in such cases the milder preparations will be found advantageous.

The third, and perhaps the most efficacious, form in which mercury may be used, is that of the bichloride, one of the most valuable medicines of the entire Pharmacopœa. "A treatise might be written on the virtues of this remedy, and the vast field of disease over which it exercises a sanative influence. Combined with Peruvian bark—which the chemists say is incompatible, but the product of the decomposition said to be produced by which, may be the very substance which acts most beneficially—it is almost a panacea for most of the strumous inflammations in children and young people; and its power in controlling scrofulous ophthalmia, corneitis, and iritis, &c., extends equally to the cure of kindred affections in the ear. It is the best remedy I know of for inducing absorption of lymph deposits in the membrana tympani, and general thickening and opacity of that structure, as well as very old cases of chronic inflammation of the membrane of the cavitas tympani. It is, moreover, when properly administered, one of the safest as well as the surest preparations of mercury; it may be taken for a great length of time; it seldom interferes with the ordinary occupations or amusements of the individual; it leaves no ill effects; it rarely induces ptyalism; and patients improve in health, and absolutely grow fat while using it. It may be given alone, either in pill or dissolved in nitrous ether, proof spirits, or some of the tinctures, such as cascarilla, but it is much more soluble in distilled water than is generally known; it may be combined with the muriated tincture of iron with good effect, or with some of the preparations of sarsaparilla; but bark—either the tincture, syrup, or decoction—is of all others the medicine best suited for its administration. Our Dublin preparation of the syrup is, particularly for children, a good vehicle for it, provided the mineral is first dissolved in a little distilled water. Oxymuriate of mercury and bark sometimes disagree, producing, shortly after being taken, pain in the stomach, tenesmus, griping, and even diarrhœa; in such case it will generally be found that it was taken before breakfast or on an empty stomach; it should therefore be administered an hour or two after meals. But when it disagrees, even with such precautions, a separation of the constituents will obviate the unpleasant effects; thus the mercury may be taken an hour or two before or after the bark. From the sixteenth to the eighteenth, or even a quarter of a grain, may be taken three times a day, according to the circumstances of the case, for weeks and even months together, with, however, short intervals occasionally. The preparations of iodine and potassium may be employed in aural affections, and will be found efficacious just as they act on the general health or the diseases of other organs; so likewise with cod-liver oil. The only medicine I know of which appears to exercise an influence upon tinnitus is leopard's bane, the arnica montana."

*The Statistics and Nosology of Diseases of the Ear* are discussed in chapter iii., and although one of the most instructive and interesting chapters in the work, our space forbids any but a very brief analysis of its contents:

"Out of the 2385 cases recorded we perceive that 579 were simply cases of impaired hearing produced by impaction of the external auditory passage with cerumen; 114 of so-called nervous deafness; 25 of tinnitus aurium, unaccompanied at the time by deafness or any apparent disease; 14 of otalgia; 7 of deaf-dumbness, either congenital or acquired; 2 of accidental hemorrhage from the tympanal cavity; 7 of congenital malformation; 20 of collapsed membrana tympani; and 2 of tumors of the auricle:—making in all but 770 cases of diseases of the ear not directly traceable to inflammation or its effects."

"Diseases of the auricle and external meatus amount to nearly one-half of the entire; affections of the membrana tympani, exclusive of collapse, number 819, or nearly one-third of the entire; and diseases of the middle ear amount to 101, or about a twenty-third of the whole. The term *otitis* is here applied solely to inflammations of the cavity of the tympanum; but as it is not possible to limit inflammatory action to the peculiar structure in which it is originally set up, we may suppose that a large proportion of the diseases registered as affections of the external drum-head must have extended sooner or later to the internal surface of that membrane, and the investitures of the cavity of which it forms the outer boundary."

Chapter IV. is devoted to the description of the anatomy of the auricular region; the auricle and external auditory canal; wounds and injuries of the auricle; cutaneous affections, &c., &c.

Eczema and herpes are not only much the most frequent forms of skin disease in the auricle, but may, if allowed to extend into the meatus, produce disease on the external layer of the tympanum and deafness.

*Eczema aurium* "principally occurs in females of middle and advanced life; but it also happens to children from six to twelve years of age. In the latter, however. it is of much more active nature, at the same time that it is much more amenable to treatment. In young persons the eruption often co-exists with scald head, and in both young and old, if the disease is allowed to exist for any length of time, it extends into the meatus, and even over the surface of the tympanal membrane, which it thickens and renders opaque. In old persons a collection of branny scales accumulates in the external tube; and in young persons a thick creamy discharge coats over the lining of the canal and the external layer of the membrana tympani.

"Cleanliness and attention are indispensable to the eradication of these affections. In the first instance, continual poulticing with any emollient substance which keeps up heat and moisture is necessary. Linseed meal, boiled bread and milk, or well mashed turnips, will be found useful applications. Afterwards, when the extreme heat, swelling, vesication, and redness, have subsided, a solution of the liquor plumbi, in the proportion of a drachm to the ounce, applied with several bits of fine lint, so as completely to envelope the auricle, and the evaporation prevented by covering over the whole with a piece of oiled silk, rarely fails to lessen the irritation, and reduce the parts to a healthy condition. The solution of gutta percha in chloroform, lately introduced by Dr. Graves in the treatment of other skin diseases, I have found a very admirable remedy in the chronic form of eczema aurium. The part should be painted over several times, until a complete varnish has been laid on, when the greatest relief from the heat and itching is experienced. The application should be repeated from day to day, as the material soon begins to peel off, but should never be applied until the acute attack has subsided. When the auricle is shining, of a bright red, and swollen, punctures made with the point of a lancet, particularly in the helix, will give great relief. In the chronic stage good may be effected by painting the part with a strong solution of nitrate of silver.

"But while we employ these local measures, we must not neglect constitutional means. Strict attention to diet should be enforced; salt meats, savoury dishes, and pastry, ought to be avoided, and a sufficient quantity of fresh vegetables should be consumed at dinner. After the patient has been well purged, a course of Plummer's pills may be prescribed with advantage—at least five grains daily for an adult; and, in a little time, some of the preparations of sarsaparilla administered in lime water will hasten the cure, and assist to eradicate the disease from the system. This affection is very apt to relapse, and we should, therefore, continue both our local and constitutional remedies long after the inflammatory symptoms have subsided. Old ladies think they never can have a sufficient amount of warmth about the head, and it is very difficult to induce them to leave off even one flannel nightcap; but we should at least make the attempt, as the head and ear ought to be kept as cool as possible. As the swelling and inflammatory symptoms subside, we should again turn our attention to the state of the auditory tube. If any discharge exists, the meatus should be syringed gently with tepid water daily; and both it, the concha, and the tympanal membrane, washed over every second or third day with a solution of nitrate of silver of the strength of at least twelve grains to the ounce. Still more advanced in the progress of the treatment, when the exudation has completely ceased, and the thickened cuticle has been quite removed, much benefit will be derived from smearing over the tube and membrana tympani with brown citrine ointment (*Ungt. Citrinum Fuscum*) every third or fourth day. It should be applied in a melted state with a camel's-hair pencil, and diluted by about one-third of almond oil. This ointment, in which I have great faith in all diseases similar to that now under consideration, should be made with either rape-oil or cod-liver oil, instead of the olive oil with the lard or butter usually directed in the Pharmacopœas; it is then of a much darker color, and never becomes hard or crumbly."

Mr. Wilde specifies six kinds of postaural tumors, some of which may prove fatal in either the acute or chronic form.

The first generally occurs in young females—is the result of disease in the small gland lying on the mastoid process immediately above the insertion of the sterno-mastoid muscle—sometimes acquires the size of an almond—and is relieved by the external application of iodine and the internal exhibition of tonics, particularly iron. Cure always tedious.

The second is simply a suppurating gland—generally observed in scrofulous constitutions; not unfrequently met with in children during dentition—the great bulk of the gland is below the level of the external meatus and this is a diagnostic of some importance, as a very formidable and often fatal swelling, which sometimes occurs behind the auricle, is always seated higher up. This form of tumor is in most instances removed by fomentations, poultices, aperient medicines—simple dressings to the sore, bark mixture and country air.

The third form of tumor has presented itself but twice to Mr. WILDE. It is a chronic abscess, very similar to lumbar abscess and like it appears to be connected with diseased bone. It is painless, fluctuating, the size of half a hen-egg, occupying all the space behind the auricle.

The fourth variety of tumor demands our special attention. It is the result of acute inflammation, either arising from periostitis of the mastoid process, and often extending over the whole parietal region; or caused by accumulations of matter in connexion with the mastoid cells, the result of disease spreading from the middle ear; or it may arise from chronic inflammation and otorrhœa producing caries. As soon as the case presents itself the tumor should be immediately opened by a free incision carried down through the periosteum to the bone.

True aneurism of the posterior aural artery constitutes the fifth form of tumor.

The sixth tumor which occurs in this region is a malignant fungus, of which our author has seen three cases.

*Foreign bodies* often get into the meatus. Rude efforts made to extract them are as likely to cause mischief as the bodies themselves. Syringing the meatus with plain warm water assisted by the dexterous use of the forceps or curette is both the simplest and most effectual means of removing the substance if it be small, whether it be an insect or a pebble.

Impaction of the auditory canal with hardened wax is the cause of one of the most common and curable forms of deafness. The diagnosis is made through the aid of the speculum—the treatment consists in its removal by syringing and the use of the spatula and forceps. If the patient experiences much pain from our manipulation it is better to desist, and drop a little warm oil into the ear once or twice a day, or keep a bit of cotton moistened with oil in the passage, until the wax has been partially softened.

*Abscess in the external meatus* is one of the most painful and frequent inflammations of the external ear. It is analogous to a stye upon the eye-lid, but owing to the structure in which it is placed, it is far more painful.

To prevent suppuration the solid nitrate of silver used so as to blacken the skin is the most efficacious local application. When matter has formed and neared the surface—not however, till then, the abscess should be opened with a double edged-knife. At the same time much relief will be afforded by fomentations and poultices and the steam of hot water. Careful constitutional treatment is also specially indicated.

"*Diffused inflammation of the external meatus* is a matter of much more serious consequence than either the profession or the public are aware ; for, frequently as it occurs, and lightly as it is treated, it generally ends in the establishment of a disgusting disease—otorrhœa, which always impairs the hearing, oftentimes leads to total deafness, and, in some cases, ends in death. Yet how frequently do we hear practitioners speak of the patient having "only a slight discharge from the ear." At times the symptoms of inflammation of the lining of the meatus are so slight, and produce such litte uneasiness, that the patient first becomes conscious of his disease by feeling something wet in his ear, when upon applying the finger, or a towel, he discovers that a discharge of thin, whitish, muco-purulent matter has been established ; or in infants and young children, about the period of dentition, the nurses and attendants observe the flow of matter as the earliest symptom of the disease. This is the subacute or *catarrhal inflammation* of the dermis, and the external layer of the membrana tympani, which is always attended with otorrhœa, and which frequently remains in a chronic condition for years. The state of the external aperture on the first onset of the disease, and before it has become thickened or excoriated by the discharge, is normal ; but within the cuticle is white, pulpy, and detached, and the skin beneath it is usually of a pinkish color. This is a disease of infancy and youth, and is one of the most decidedly strumous affections with which I am acquainted."

Cutaneous diseases of the meatus—morbid growths and malignant diseases, &c., we shall not stop to notice.

Chapter V. contains a description of the anatomy and diseases of the membrana tympani. The congenital malformations, wounds and injuries of this structure need not detain us. Not so however, with its inflammations.

It is only within the last few years that *myringitis*, or inflammation of the membrana tympani, has been recognized or described by authors ; and Mr. WILDE says its varieties, with their peculiar symptoms, are by no means accurately understood.

In acute myringitis the vascularity generally occupies the true fibrous structure and is usually the result of cold, sudden exposure to a low temperature, foreign bodies &c. The auditory canal, the mucous membrane lining the tympanum, the investitures of the eustachian tube, the labyrinth, the internal ear and the auditory nerve itself, will sooner or later participate in the unhealthy action going forward. The alterations may result in simple loss of hearing, or may prove dangerous to life, through an extension of the inflammation to the bones, the membranes of the brain, or the encephalon itself. The symptoms are local and constitutional.

The patient is generally seized with sudden and intense pain in the ear, usually making its first appearance at night. The pain is excruciating—likened to that of a sharp instrument penetrating through the ear to the brain. It is aggravated by coughing, sneezing, chewing, swallowing and by pressure on the tragus. There is also pain and soreness over the side of the head, in the teeth, in the temple, in the eye, and behind the jaw. If not relieved by treatment, the pain extends to the throat and mastoid region—and is increased by pressing the mouth of the eustachian tube with the finger. "The severity of the pain experienced, and the extent of soreness to the touch, is to a certain degree a test of the amount of inflammation; and the peculiarity of the pain is also a means of judging of the seat of the inflammation; for if it is experienced in swallowing, mastication, or sneezing, &c., we may presume that the inflammation has extended over the middle ear."

Ear-ache is often considered a *neuralgic* affection and treated accordingly,—by pouring into the ear the various nostrums, sedatives and stimulants, calculated to allay pain in external parts. Mr. Wilde, with all his vast opportunities for observation, has never met but two instances of so called "nervous otalgia" for which he could not discover some direct *visible* cause; and the celebrated Berlin aurist, Dr. Kramer, has never seen ear-ache without evidence of inflammation either of the meatus or membrana tympani.

*Tinnitus aurium* and deafness, either partial or total, of the affected side, come on cotemporaneously with the pain, or succeed it in a few hours after.

The constitutional symptoms briefly are symptoms of catarrh, headache, sometimes hemicrania, heat of surface, anxiety of countenance, sleeplessness, restlessness; in severe cases accelerated circulation, occasional rigors, and sometimes delirium. The digestive organs are not much affected; the urine is high colored, and, towards the termination of the acute symptoms, deposits a copious pinkish sediment.

The physical signs, in severe cases, are pain, heat and a slight erysipelatous blush of the auricle; œdema, pain, fullness and heat over the mastoid region; soreness of the scalp on the affected side.

Our limits forbid our enumerating the appearances of the parts as revealed by the speculum.

Acute myringitis chiefly attacks the young and middle aged; persons with light hair and fair skin are more subject to it than brunettes, and one ear is more frequently affected than both. "So much more frequent are its attacks in spring than at any other period of the year, that it sometimes seems to be epidemic at that time." In regard to the treatment, our author says he has never had occasion to employ general blood-letting; cupping or leeching are invariably necessary; leeches are best and the relief afforded by them is often instantaneous and always most decided.

The temperature "should be strictly attended to; the patient should, if possible, be confined to a warm and well ventilated apartment, or, if obliged to go abroad, the cold air should be carefully excluded from the ear; but in the severe form of the disease it is absolutely necessary to confine the patient to bed."

Great comfort will be afforded by the employment of moist heat. When there is much tenderness to the touch or soreness or pain over the side of the scalp or face, or about the external ear, the various sedative applications are called for. Tincture of aconite, belladonna with the compound camphor liniment and chloroform are esteemed the best by Mr. WILDE.

D. W. Y.

[*To be Continued.*]

